# Feasibility of three wearable sensors for 24 hour monitoring in middle-aged women

**DOI:** 10.1186/s12905-015-0212-3

**Published:** 2015-07-30

**Authors:** Jennifer Huberty, Diane K. Ehlers, Jonathan Kurka, Barbara Ainsworth, Matthew Buman

**Affiliations:** School of Nutrition and Health Promotion, Arizona State University, Phoenix, AZ USA

**Keywords:** Objective measurement, Sleep, Health behaviors, Physical activity

## Abstract

**Background:**

The purpose of this study is to determine the feasibility of three widely used wearable sensors in research settings for 24 h monitoring of sleep, sedentary, and active behaviors in middle-aged women.

**Methods:**

Participants were 21 inactive, overweight (*M* Body Mass Index (BMI) = 29.27 ± 7.43) women, 30 to 64 years (*M* = 45.31 ± 9.67). Women were instructed to wear each sensor on the non-dominant hip (ActiGraph GT3X+), wrist (GENEActiv), or upper arm (BodyMedia SenseWear Mini) for 24 h/day and record daily wake and bed times for one week over the course of three consecutive weeks. Women received feedback about their daily physical activity and sleep behaviors. Feasibility (*i.e.*, acceptability and demand) was measured using surveys, interviews, and wear time.

**Results:**

Women felt the GENEActiv (94.7 %) and SenseWear Mini (90.0 %) were easier to wear and preferred the placement (68.4, 80 % respectively) as compared to the ActiGraph (42.9, 47.6 % respectively). Mean wear time on valid days was similar across sensors (ActiGraph: *M* = 918.8 ± 115.0 min; GENEActiv: *M* = 949.3 ± 86.6; SenseWear: *M* = 928.0 ± 101.8) and well above other studies using wake time only protocols. Informational feedback was the biggest motivator, while appearance, comfort, and inconvenience were the biggest barriers to wearing sensors. Wear time was valid on 93.9 % (ActiGraph), 100 % (GENEActiv), and 95.2 % (SenseWear) of eligible days. 61.9, 95.2, and 71.4 % of participants had seven valid days of data for the ActiGraph, GENEActiv, and SenseWear, respectively.

**Conclusion:**

Twenty-four hour monitoring over seven consecutive days is a feasible approach in middle-aged women. Researchers should consider participant acceptability and demand, in addition to validity and reliability, when choosing a wearable sensor. More research is needed across populations and study designs.

## Background

The role of moderate-to-vigorous physical activity in chronic disease prevention is well established. More recently, the combination of the roles of sleep [1, 2], sedentary [3, 4], and even light intensity behaviors [5, 6], for chronic disease prevention have underscored the need to understand the full 24 h period and how these behaviors can be harnessed for health promotion [7]. The use of wearable sensors (*e.g.*, accelerometers) to monitor these behaviors in surveillance, epidemiological, and intervention studies has steadily grown in both the physical activity [8] and sleep fields [9]. A recent integration of technologies and the availability of commercial sensors have triggered a shift toward 24 h monitoring that allows concurrent assessments of sleep, sedentary, and active behaviors. However, to date we are not aware of any studies that have explored the feasibility of a 24 h approach to behavioral monitoring and factors that influence the user experience, including sensor placement, sleep/wake transitions, and behavioral feedback that may impact assessment.

Information about best practices for behavioral monitoring and factors that influence the experience of the user are limited. In a thorough review of best practices for accelerometer use in physical activity, Ward and colleagues (2005) provided information about appropriate sensor selection, quality and dependability, sensor use protocols, sensor calibration, analysis of accelerometer data, and integration with other data sources [10]. However, no information was presented about the feasibility of wearing the sensors (*e.g.*, comfort of sensors, preferred placement) [8]. The National Health and Nutrition Examination Survey (NHANES) in 2011 switched from a waist worn accelerometer to a wrist worn accelerometer partially because researchers believed the wrist location would improve participant compliance. Researchers have noted enhanced adherence with the move to this location [11]. A few studies have reported participant-based strategies (*e.g.*, participants perform tasks to promote compliance) or investigator-based strategies (*e.g.*, activities performed by researchers to help participants wear the sensors) to help improve wear-time compliance, but none have explored or reported feasibility [12, 13].

Researchers have yet to explore if wearable sensors are acceptable (*i.e.*, is the tool suitable or attractive?) and demanded (*i.e.*, is the tool likely to be used?) by the participant [14]. A recent review documents differences in compliance between hip and wrist protocols and cites other studies using wrist protocols that may provide comparative data for wear time [11]. Determining the feasibility of wearable sensors may have important implications for the validity of data collected in terms of compliance and potential disruption in normal activities. Additionally, this information may inform the design of behavioral interventions, development for future wearable sensors, and contribute to best practices for behavioral monitoring [9, 15, 16].

Middle-aged women (30–64 years of age) are one of the most inactive populations in the United States [17]. Middle-aged women are also twice as likely to have disturbed sleep compared to men due to the menstrual cycle, pregnancy and postpartum periods, and menopause. In the interest of designing interventions to improve sleep, sedentary, and active behaviors in middle-aged women, there is a need to explore the feasibility of wearable sensors in this population.

The purpose of this study was to determine the feasibility (*i.e.*, acceptability, demand) of three widely used wearable sensors in research settings; ActiGraph GT3X+ accelerometer (Actigraph, Pensacola, FL), GENEActiv accelerometer (Activinsights, Kimbolton, Cambridgeshire, UK), and SenseWear Mini Armband (BodyMedia, Inc., Pittsburgh, PA), for 24 h monitoring of sleep, sedentary, and active behaviors in middle-aged women.

## Methods

### Participants

The Institutional Review Board at Arizona State University approved the study. Women were recruited with posted fliers, word of mouth, through local libraries, listservs, and a newsletter. Women were eligible to participate if they were insufficiently active (not meeting regular physical activity guidelines of at least 30 min of moderate intensity activity five days per week [18, 19]), willing to wear a wearable sensor, and between the ages of 30 and 64 years. Women were screened for eligibility by telephone when they contacted the research team about the study.

### Procedures

Eligible women participated in a baseline appointment in which they reviewed and signed a written informed consent form, completed a demographic questionnaire, and received the first of three sensors, including instructions for wear (Table 1). The three sensors included were: ActiGraph GT3X+ accelerometer, GENEActiv accelerometer, and SenseWear Mini Armband. Women did not wear all three sensors simultaneously. The order in which women received each sensor was randomly assigned and counterbalanced to control for ordering effects. Research team members met with women face-to-face at locations convenient for each participant (*e.g.*, coffee shops, women’s homes or workplaces, the researchers’ office) to distribute and retrieve sensors, give wear instructions and feedback on daily physical activity and sleep behaviors, and administer satisfaction surveys (after each sensor) and an interview (last meeting only). The physical activity and sleep data included on the feedback reports from each sensor are listed in Table 1. A researcher discussed the results with participants during the scheduled appointments.

**Table 1:** Specific instructions and feedback provided across three wearable sensors

	Actigraph GT3X+	GENEActiv	SenseWear Mini
Placement	Non-dominant hip along the anterior axillary line	Non-dominant wrist with watch face facing upwards	Facing upwards on backside of non-dominant upper arm
Sleep	Move monitor from hip to non-dominant wrist (strap provided) at bed time	Press button on watch face to record wake and bed times	None
Waterproof	Remove monitor before water-based activities and bathing	Monitor is waterproof - wear for 24 h per day	Remove monitor before water-based activities and bathing
Participant log	Record daily wake time, bedtime, and periods of non-wear. Wake time was defined as getting up for the purpose of being up for the day. Bedtime was defined as going to bed for the purpose of sleeping.		
PA feedback	Daily Sedentary, Light, Moderate, Vigorous activity; Steps	Daily Sedentary, Light, Moderate, Vigorous activity	Daily Sedentary, Light, Moderate, Vigorous activity; Steps; Energy Expenditure
Sleep feedback	Total Sleep Time; Sleep Onset Latency; WASO; Sleep Efficiency	Total Sleep Time; Sleep Onset Latency; WASO; Sleep Efficiency	Total Sleep Time

*WASO* Wakefulness After Sleep Onset. Only total sleep time was provided for the SenseWear Mini because it has not been established as a valid and reliable measure of sleep quality

Days on which an appointment was scheduled before 10:00 am were considered Day 1 of 7 of the data collection period, while days on which an appointment was scheduled after 10:00 am were considered Day 0 of 7 days. This scheme was used to ensure the first day of each data collection period provided the opportunity for at least 10 h of wear time during the participant’s wake period.

### Measures

Specific instructions related to each sensor were provided (Table 1) and women were instructed to wear each sensor for 24 h per day for seven consecutive 24 h periods on their non-dominant hip/wrist (ActiGraph), wrist (GENEActiv), or upper arm (SenseWear). A seven-day sampling period was selected to follow best practices for sleep and activity monitoring [20, 21]. Sleep and wake windows were determined for each sensor using reported bed and wake times recorded by the participant on a daily log. Women were also asked to record any periods of non-wear on their daily log. The placement of each sensor was determined by examining the literature and choosing the location where the best validation data were available for each sensor and behavior (*i.e.*, physical activity, sleep).

### ActiGraph GT3X+

The ActiGraph GT3X+ is a small battery-operated electronic motion solid-state sensor (micro-electromechanical systems) designed to measure the rate and magnitude of body movement in a vertical and horizontal plane (accelerations). Women were instructed to wear the ActiGraph GT3X+ sensor on their non-dominant hip with an elasticized waist belt during the day, move the sensor to their non-dominant wrist using a wrist strap at bedtime, and return to the waist upon awakening. These procedures were adopted given the current lack of acceptable algorithms to process ActiGraph data on the wrist. Women were also instructed to remove the sensor during water-related activities (*e.g.*, showering, swimming). Sensors were initialized to sample movement at 40hz and raw data were aggregated into 60-s epochs. After filtering out sleep windows, wear time was determined by identifying periods of non-wear when ≥60 consecutive “zero” intensity counts were present. For behavioral feedback, the Freedson algorithm [22] was used to quantify wake periods and the Sadeh algorithm [23] was used to quantify sleep periods. All data were processed in Actilife 6.10.0 (Pensacola, FL).

### GENEActiv

The GENEActiv is a lightweight, waterproof micro-electromechanical sensor that utilizes selectable frequency-based (10-100 Hz) raw, waveform data collection. Women were instructed to wear the GENEActiv sensor on their non-dominant wrist throughout the entire 24 h period, including water-based activities. Sensors were initialized to sample movement at 40hz and raw data were aggregated into 60-s epochs for wake and sleep periods [24]. After filtering out sleep windows, non-wear periods were defined as ≥60 consecutive epochs with a 20-epoch forward-moving standard deviation ≤ 0.05. This method was used in lieu of the zero-count method because the GENEActiv does not register absolute zero counts when the unit is not in motion (verified by comparing to wear-time logs of participants). For behavioral feedback, the Esliger algorithm [25] was used to quantify wake periods and an excel macro provided by the manufacturer and consistent with the Sadeh algorithm [23] was used to quantify sleep periods. All activity data were processed in SAS 9.3 (Cary, NC).

### SenseWear Mini

The SenseWear Mini Armband is a triaxial accelerometer that measures electrical conductivity of the skin, the body’s surface temperature, and heat flux. Women were instructed to wear the SenseWear Mini on their non-dominant upper arm throughout the entire 24 h period and remove only for water-based activities. Sensors were initialized with biometric information including date of birth, gender, height, weight, and handedness of the participant. The SenseWear Professional 7.0 (Pittsburgh, PA) software provided proprietary estimations of “on-body time” (used to determine wear time), activity and energy expenditure classifications of wake periods, and sleep estimations [26, 27].

### Data analysis

Acceptability was defined as the extent to which each wearable sensor was acceptable to participants [14]. Acceptability was assessed with a satisfaction survey completed after wearing each sensor and included items addressing perceived ease of wear, satisfaction with the sensor placement, comfort during sleep, length of the sampling period, perceived inconvenience completing the daily log, and reasons for non-wear. Response options were on a 5-point Likert-type scale and were collapsed to reflect positive (strongly agree and agree) *vs.* neutral (neither agree or disagree) *vs.* negative (strong disagree or disagree) valences. Results of the satisfaction surveys were summarized using descriptive statistics.

Demand was defined as the extent to which each sensor was used. Demand was measured using wear time estimates, percentage of wake periods with valid data (*i.e.*, worn ≥10 h per day), and percentage of sleep periods with valid data (*i.e.*, worn during sleep). One-way repeated measures analysis of variance (ANOVA) was used to examine differences in wear time, total bedtime, valid wake periods, and valid sleep periods across sensors. Differences were considered significant at *p* < 0.05. Analyses of the wake period (wear, non-wear, valid days) included Days 1 through 7, while analyses of the sleep period (total bed, valid nights) included Days 2 through 7 only. Some women met with the research team on the morning of Day 1, permitting this day as an eligible day for the assessment of physical activity. However, this eliminated Day 1 as an eligible day for the calculation of total bedtime due to missing sleep data from midnight until enrollment in the study.

To gain greater insights into women’s perceived acceptability and demand of the wearable sensors, women were asked to participate in a short interview to compare and contrast their experiences across sensors. Interviews were transcribed verbatim and analysis was conducted using NVivo 10 (QSR International, 2012). The lead researcher read each transcript and developed initial codes using an inductive process [28]. After initial coding, the themes were refined after a second reading and a codebook was created. A second researcher reviewed the data to confirm the coding. Discrepancies in coding between researchers were discussed until consensus was achieved.

## Results

A total of 83 women contacted the research team with interest in participating in the study. Of those, 23 were ineligible, 37 never followed up or were not interested after expressing initial interest, 23 enrolled, and 21 completed the study. Participants were 21 inactive women aged 30–64 years (*M* age = 45.31 ± 9.67), overweight (*M* BMI = 29.27 ± 7.43), Caucasian (95 %), and college educated (74 %).

### Acceptability

Comparisons between wearable sensors and satisfaction survey responses are presented in Table 2. Women felt the GENEActiv and SenseWear Mini were easier to wear and preferred the placement compared to the ActiGraph. They also rated the GENEActiv and SenseWear Mini as most comfortable during sleep. Notably, the majority of women felt that neither the 7 days of monitoring nor the 24 h of monitoring was too long. Women also did not think that completing the daily log was inconvenient. Findings from the interviews and information from the surveys specific to individual sensors are reported below.

**Table 2:** Satisfaction survey results: percentage of women who positively endorsed* acceptability items by wearable sensor

	Actigraph GT3X+		GENEActiv		SenseWear Mini	
	N	(%)	N	(%)	N	(%)
N	21		19		20	
Device acceptability						
Easy to wear	9	(42.90)	18	(94.70)	18	(90.00)
Placement	10	(47.6)	13	(68.4)	16	(80.0)
Comfortable during sleep	14	(66.7)	15	(78.9)	17	(85.0)
7 Days too long	7	(35.0)	6	(31.6)	8	(45.0)
24 h each day too long	8	(38.1)	7	(36.8)	8	(45.0)
Log was inconvenient	4	(21.1)	5	(26.3)	6	(30.0)
Behavioral feedback content						
Moderate-vigorous physical activity feedback	21	(100.0)	15	(83.3)	20	(100.0)
Light intensity activity feedback	18	(90.0)	15	(88.2)	17	(85.0)
Sedentary activity feedback	17	(85.0)	15	(83.3)	18	(90.0)
Sleep quantity/quality feedback	19	(100.0)	16	(100.0)	16	(84.2)

*Positively endorsed = women agreed or strongly agreed

#### Preferred sensor

A majority of women preferred the SenseWear Mini. One woman said, “That was the one you could hide the most so I liked it for that reason.” Another woman said, “The armband, I never had to think about it once it was on and I liked the beeps that were reassuring that it was working.”

#### Least preferred sensor

Both the ActiGraph and GENEActiv were equally reported as least preferred in the interviews. The ActiGraph was least preferred mostly due to a lack of comfort and ease of use. A woman shared, “That you had to take it apart and put in on your arm at night . . . I just didn’t like it around my waist partly because I was never sure I had it in the right location. And if you’re not skinny then it’s cumbersome.” Another woman said, “I didn’t like where it sat on my body.” “The belt part would slide around . . . and just under your clothes and it was a problem,” said another woman.

The GENEActiv was also reported as a least preferred sensor due to its appearance (*i.e.*, bulky, looks like watch but doesn’t function like a watch), lack of comfort and lack of ease of use. One woman said, “I couldn’t wear a regular watch with it, it didn’t tell time, and it was not very pleasing to the eye.” Another woman reported, “It just constantly got in the way with the dealing of life.” However, according to the satisfaction surveys, the GENEActiv was the easiest to wear and a majority liked the placement (Table 3).

**Table 3:** Response rates for number of valid wake time and time in bed by device

	Wake time					Sleep time				
Device	Valid days^a^ (%)					Valid nights^b^ (%)				
	≥1	≥4	7	Mean	(SD) days	≥1	≥4	6	Mean	(SD) days
Actigraph	100	100	61.9	6.57	±0.75	100	100	85.71	5.81	±0.51
GENEActiv^c^	100	95.2	95.2	6.76	±1.09	100	95.24	90.48	5.71	±1.10
SenseWear^d^	100	100	71.43	6.52	±0.93	100	90.47	80.95	5.62	±0.92

^a^A valid day was defined as having 10 or more hours of wear time during waking hours

^b^A valid night was defined as wearing the monitor during the self-reported sleep period

^c^One device malfunctioned after Day 2, Night 1. If excluded, 100 % of participants wore the monitor for all 7 days and 95 % for all 6 nights

^d^One device malfunctioned after Day 4, Night 3. If excluded, 75 % of participants wore the monitor for all 7 days and 85 % for all 6 nights

#### Most convenient location of the body

Many of the women felt that the upper arm (SenseWear Mini) was the most convenient location to wear a sensor. Women preferred the placement of the SenseWear as compared to the other wearable sensors (Table 3). Women said the armband, “was out of sight, out of mind,” “was more hidden and underneath my shirt,” “was above and out of the way. I mean I didn’t have to think about it very much,” and, “was more acceptable in society, people aren’t going to question you, ‘What is that?’”.

#### Least convenient location of the body

The least convenient location to wear a sensor was the waist (ActiGraph). Women reported that the belt took some getting used to, that it moved up and down depending on what they were doing and the belt was uncomfortable. One woman said, “It was cumbersome, didn’t always fit under clothes and unless you are skinny where are your hips?” Another woman reported, “Depended on the clothes you wear and when you go to the bathroom.” According to the satisfaction survey, the waist was the least accepted location to wear a wearable sensor (Table 3).

#### Comfortable body location

More women reported that the upper arm (SenseWear Mini) was the most comfortable location for the wearable sensors as compared to the wrist and waist. Women said the sensor on their upper arm “didn’t bother me and it was just there,” and “for the most part I didn’t feel it.” However, a few women reported liking the wrist location. One women said, “The wrist because I usually wear a watch so I’m used to having something around that part of the arm.” Another woman stated, “I usually wear something on my wrist so it wasn’t a big change…I always wear bracelets.” Most women agreed that all three wearable sensors were comfortable during sleep (Table 3).

#### Least comfortable body location

Women reported that the waist was the least comfortable body location. “It was an annoying location relative to your clothes,” reported one woman. Another said, “When I ran it was hard to keep it from moving.” Findings from the satisfaction survey were similar (Table 3).

#### Biggest motivators and barriers to wearing sensors

Women most often reported the feedback they received from researchers as their biggest motivator to wearing the sensor. Specifically, they liked receiving a daily breakdown of their activity levels and sleep quality. Barriers to wearing the sensors included appearance, comfort, and inconvenience. Specifically, even though women most preferred the armband and its location, many of the referrals to appearance were in relation to the armband. One woman said, “The attire was a big [barrier] like wanting to wear a sleeveless dress or something and having the armband on.” Another woman said her barrier was “how [the instrument] would go with whatever I had to wear.” Women also mentioned that the comfort of the wearable sensors was a barrier. Comfort became a barrier for some women during exercise and hot weather. Women also reported that if wearing the sensor was inconvenient (*i.e.*, had to take it on and off a lot) they may have forgotten to wear it. According to the satisfaction surveys, women reported it was easy to remember to take off the SenseWear Mini and ActiGraph before water-based activities (95 and 81 % agreed, respectively). However, fewer women thought that it was easy to remember to put the sensor back on afterwards (70 and 62 % agreed, respectively). The GENEActiv in particular was mentioned as inconvenient because it got in the way of “giving the kids a bath or doing the dishes . . . just in the way.” When asked if they liked that the GENEActiv was waterproof and did not have to be removed, 63.2 % of women agreed.

#### Preferred sensor to wear long-term

When asked which sensor women would prefer if asked to wear one long-term (*e.g.*, 24 h a day for a month), women said they would prefer the SenseWear Mini. One woman said, “It would be the armband. It’s lighter, not as bulky, and not in the way. I don’t have things catching on it or getting on it all the time.” One woman stated that even though she felt “dorky” wearing the armband, “It gave me the best feedback that I really liked. The reassuring beeps, the most comfortable, and didn’t ever get in the way of taking care of baby and life and that sort of thing.”

#### Informational feedback most valued

Women really valued the informational feedback that they received. All women agreed that they liked receiving physical activity information from the ActiGraph and SenseWear, and most agreed in relation to the GENEActiv (Table 3). However, during the qualitative interviews more women reported that the sleep information was more beneficial as compared to the physical activity information. Women felt this way because they were “curious” about their sleep and hadn’t had the opportunity to “look” at it. Women reported, “I liked the sleep information because it validates that I don’t sleep that well,” and “I was really interested in the sleep stuff because I was curious why. I figured I have, like, sleep issues, so now this is just confirming it.”

Even though more women valued the sleep information, most women reported that the physical activity feedback increased their awareness about their physical activity behaviors. Many said it was a “wake-up call.” One woman admitted, “I was stunned at my personal level of activity or lack thereof.” Another woman said she didn’t realize how much of her time was spent sedentary. “I was really surprised with the amount of [my] physical or moving on a daily basis . . . showing sedentary or light activity was disappointing more than anything.”

#### Changes to current behavior

When women were asked if they planned on using any of the information they received to make any changes in their behaviors, women reported being more aware of their activity levels, eating better, and establishing goals for exercise. One stated she would go to the doctor about her sleep behaviors. Women said, “This is me thinking about [physical activity] way more than I had before and how to fit it into my lifestyle right now,” and “I certainly can see that I can make a little more effort to do some moderate to light exercise, even taking a walk.” One woman said, “Definitely prompted me to eat better.” Another woman established daily step goals, “I’m not at 10,000 steps yet, but if I’m making daily changes then I should be able to get to a better place.”

#### Demand

Table 3 presents rates of valid wake time and sleep time for each sensor. No significant differences in the percentage of valid days, *F*(2,40) = 2.8, *p* = 0.07, or valid sleep nights, *F*(2,40) = 0.86, *p* = 0.43, were observed.

**Fig. 1:**
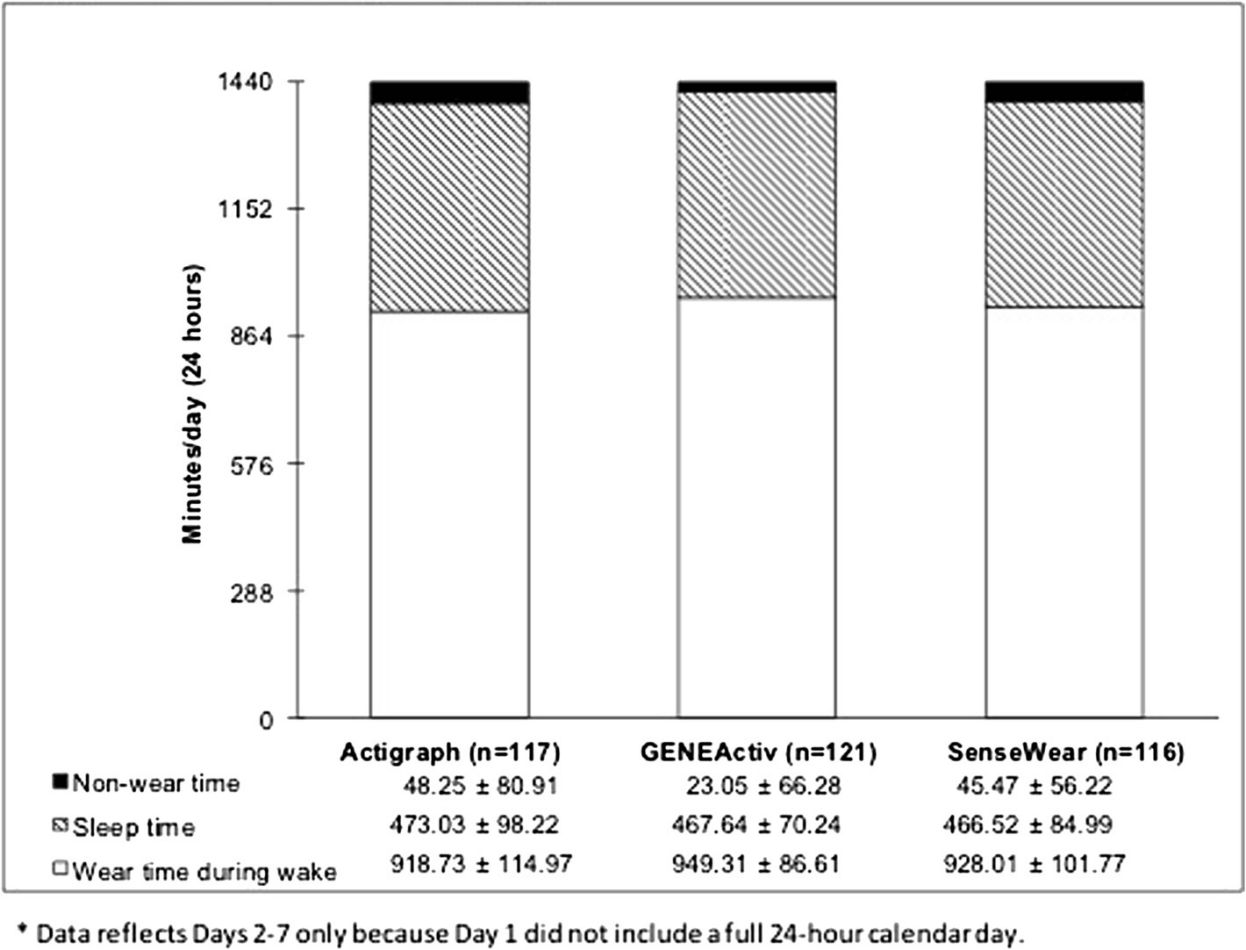
Breakdown of wear time during wake, sleep time, and non-wear time on valid days. *Data reflects Days 2–7 only because Day 1 did not include a full 24-h calendar day

There were 147 possible days of wear (7 days × 21 participants) and 126 possible nights of wear (6 days × 21 participants). Five days/nights and three days/nights were ineligible due to device malfunction for the GENEActiv and SenseWear mini, respectively. Figure 1 illustrates the breakdown of average wear and non-wear time during wake periods and average total bedtime. No significant differences in wear time were observed on valid days, *F*(2,40) = 2.87, *p* = 0.07.

## Discussion

The purpose of this study was to determine the feasibility (*i.e.*, acceptability, demand) of three wearable sensors widely used in research settings; ActiGraph GT3X+ accelerometer, GENEActiv accelerometer, and SenseWear Mini Armband, for 24 h monitoring of sleep, sedentary, and active behaviors in middle-aged women. To our knowledge, this is the first study to explore the feasibility of a 24 h approach to behavioral monitoring and factors that influence the experience of the user, including sensor placement, sleep/wake transitions, and behavioral feedback. All of the wearable sensors were acceptable and demanded by the participants. However, there were advantages and disadvantages of each sensor that may help researchers determine which sensor may be most appropriate under different conditions to measure behavior in middle-aged women.

### Acceptability

Comfort and feedback received were the two most often cited factors influencing the acceptability of the wearable sensors. The SenseWear Mini was perceived as the easiest to wear and most comfortable during wake and sleep periods. Women also preferred the placement of the SenseWear Mini on the upper arm. When asked about long-term use, most women said they would prefer the SenseWear Mini because of its comfort. Comfort and ease of wear also contributed to the acceptability of the GENEActiv. However, according to the interviews, women thought it got in the way, was bulky, and inconvenient because it did not “act” like a watch. Interestingly, the ActiGraph was least preferred due to lack of comfort, specifically the placement around the waist. ActiGraph accelerometers are some of the most commonly used sensors in physical activity research [21], and other studies have noted its acceptability among participants [29, 30]. For example, in a study by Pollard and Guell (2012), Muslim women thought the SenseWear Mini was bulky and uncomfortable, but did not report any complaints related to the ActiGraph [30].

Another major factor impacting acceptability of the sensors was auditory or visual response from the device during use. Women preferred the SenseWear Mini because it beeped when putting the sensor on and taking it off, reassuring participants that the sensor was or was not working properly. The GENEActiv and ActiGraph did not provide this type of feedback. Women specifically disliked that the GENEActiv did not provide any feedback when they pressed the event marker (*i.e.*, no beeping or lights after the watch face was pressed). In fact, women emailed the research team a number of times with concerns that they were not pressing the button or that the sensor was not working. This is in spite of women receiving detailed instructions about how to use the event marker and its purpose. Women did not comment about the lack of response from the ActiGraph. This may have been because there were no expectations about the interaction with the ActiGraph when monitoring physical activity and sleep. There is a need to explore wearable sensors that are placed on locations of the body that are “out of the way” (*e.g.*, upper arm, ankle) and provide feedback to the user about the operational status of the sensor during wear.

Women liked the informational feedback related to both physical activity and sleep. The physical activity feedback contested their perceptions of their activity levels and helped them become more aware of their daily activity. Women also expressed that they had been curious about their sleep patterns, and the feedback improved their awareness of their sleep quality. This information provides evidence that feedback about sleep, the same way that physical activity does, may motivate middle-aged women to use wearable sensors. Therefore, researchers may not only consider offering participants feedback about their activity levels before and after interventions, but may also consider including all aspects of the 24 h period to add value and improve participant compliance with data collection. This may provide an inexpensive and effective alternative to monetary incentives.

### Demand

It was notable that all sensors had remarkably high wear time. Wear time on valid days was considerably higher than reported in other studies where participants were instructed to remove the sensor at night [21]. This may indicate that, in addition to the benefit of measuring sleep behavior, 24 h monitoring may also increase daytime wear, as participants are not required to remember to replace sensors upon waking. For example, according to participant diaries in a study by Pollard and Guell (2012) taking a sensor off for sleep contributed to women forgetting to place the sensor back on the following morning [30]. It should also be noted that wear time appeared slightly higher than reported by Troiano *et al.* [11] where a 24 h wrist protocol was administered where median wear time was 22 h/day. Among the three sensors, the GENEActiv had the greatest wear time (although the differences were not statistically significant in this small sample). In fact, with the exception of one device malfunction, all participants had seven days of valid activity data when wearing the GENEActiv. This increased wear time may have been due to the fact that the sensor was waterproof and did not need to be removed for showering or swimming. While women reported they “didn’t care” that the sensor was waterproof, this may be an important consideration for selecting a sensor given its potential impact on wear time and the reduced burden for women. More research is needed in the context of interventions to determine if wearing a sensor across the 24 h period improved compliance.

### Researcher perspectives

There are a number of additional factors not addressed in this study that researchers may want to consider when choosing a sensor for 24 h behavior monitoring in middle-aged women. These factors include, but are not limited to, battery and memory capacities, raw data collection and transparency of the data, cost of the sensor and associated software, data processing needs, and availability and quality of validation data. Major lessons learned for each wearable sensor from the participant and researcher perspective are presented in Table 4.

**Table 4:** Major lessons learned for each wearable sensor

	ActiGraph GT3X+	GENEActiv	SenseWear Mini
Participant Perspective	• Least preferred	• Highest wear time	• Most preferred
	• Uncomfortable and inconvenient	• Comfortable during wake and sleep periods	• Most comfortable and convenient (*i.e.*, out of the way)
	• Difficult to remember to put back on	• Less convenient location (*i.e.*, bulky at the wrist)	• Unattractive appearance
Researcher Perspective	• Long battery life and large memory capacity	• Long battery life and large memory capacity	• Battery life 5–7 days
	• Raw waveform data collection	• Raw waveform data collection	• Raw data cannot be collected; unable to make cross-monitor comparisons
	• Semi-proprietary data processing	• Non-proprietary data processing	• Proprietary data processing
	• Less expensive monitor	• Less expensive monitor	• Most expensive monitor
	• Costs for associated software package and periodic upgrades	• Free, open-source software package	• Cost for associated software package
	• Well-developed software platform with full-range of researcher designed specifications	• Complicated, time-intensive data processing	• Easy to use software platform

### Strengths and limitations

There are two key strengths of this study. First, this study addressed feasibility of wearable sensors in middle-aged women. Second, it addressed the feasibility of measuring the 24 h period and provides data to inform the rapid growth of behavioral monitoring technologies. Despite this study’s strengths, there are limitations to be noted. First, the primary benefit of participating in this study was receiving physical activity and sleep behavior feedback, which may explain the elevated compliance observed. The feasibility of wearable sensors during an intervention-based study should be explored to further gain insight about best practices for improving participant compliance with 24 h behavioral monitoring. Second, the results do not represent the full range of feasibility concerns across all possible sensors. We did not include commercially available sensors, pedometers, or other movement-based monitors. When selecting sensors for this study, we aimed to represent measures commonly used in behavioral research and various anatomical locations. Third, our decision for participants to move the ActiGraph from hip (for wake) to wrist (for sleep) was based on the best validation data for this device. Our findings therefore may not generalize to researchers who choose use adopt a 24 h wrist protocol for the ActiGraph (as is currently being done with NHANES). Finally, this study was conducted in a small, homogeneous sample of middle-aged women, and therefore findings are not generalizable to other populations.

## Conclusions

This study reports the feasibility of wearable sensors in a sample of middle-aged women. Women reported comfort and feedback as major determinants to wearing a sensor. Findings from this study suggest researchers consider participant acceptability and demand when choosing a wearable sensor. These findings also provide valuable data to inform the development of new wearable sensors. More research is necessary in other populations and across study designs.

## Abbreviations

BMI: Body mass index
NHANES: National Health and Nutrition Examination Survey
ANOVA: Analysis of variance
